# Massive Hemoperitoneum Caused by Spontaneous Rupture of a Superficial Uterine Fundal Vein During Preterm Labor: A Case Report

**DOI:** 10.3390/jcm15010383

**Published:** 2026-01-05

**Authors:** Won-Kyu Jang, Hyun Mi Kim

**Affiliations:** Department of Obstetrics and Gynecology, School of Medicine, Kyungpook National University, Daegu 41944, Republic of Korea; cindeln@naver.com

**Keywords:** hemoperitoneum, uterine vein rupture, superficial fundal vein, preterm labor, pregnancy, obstetric emergency

## Abstract

Spontaneous hemoperitoneum in pregnancy is rare, and rupture of a superficial uterine fundal vein in an unscarred uterus is exceptionally uncommon. A 37-year-old woman at 27 + 0 weeks presented with left upper quadrant abdominal pain, and imaging revealed a localized hematoma adjacent to the left uterine fundus without active bleeding. During conservative management, she developed sudden severe pain with fetal heart rate decelerations at 27 + 6 weeks, prompting emergency cesarean delivery. Intraoperative findings showed approximately 2400 mL of hemoperitoneum caused by rupture of a superficial fundal vein, with the uterus otherwise intact, and bleeding was controlled with a fibrin sealant patch. Maternal recovery and neonatal outcome were favorable. This case underscores that rupture of superficial uterine veins should be considered in pregnant patients presenting with unexplained hemoperitoneum during pregnancy.

## 1. Introduction

Spontaneous hemoperitoneum during pregnancy is an uncommon but potentially life-threatening condition, with recent reports emphasizing its variable presentation and diagnostic difficulty [1,2]. Various causes have been described, including endometriosis-related vessel fragility, adnexal vascular rupture, and idiopathic intraperitoneal bleeding [1,3,4]. However, bleeding originating from superficial uterine vessels—particularly on the fundal serosa—remains exceptionally rare, with only isolated cases documented in the past decade [2,4].

Physiologic changes in pregnancy may contribute to venous susceptibility, including marked dilation of uterine and pelvic veins, increased venous capacitance, and elevated intra-abdominal pressure, all of which may predispose fragile vessels to rupture [3,4,5]. Although venous bleeding is low-pressure, the gravid uterus receives substantial blood flow, allowing rapid accumulation of intraperitoneal blood even from small vascular disruptions [2,5]. Recent case reports demonstrate that clinical deterioration or fetal compromise can occur abruptly, frequently requiring emergency surgical intervention [1,2,3,4].

Because of its rarity and nonspecific early symptoms, spontaneous rupture of a superficial uterine fundal vein remains a diagnostic challenge. Awareness of this condition is important when evaluating pregnant patients with unexplained abdominal pain or hemoperitoneum [1,2,3].

Despite improvements in imaging modalities, distinguishing superficial venous rupture from other causes of hemoperitoneum remains challenging, particularly in the context of preterm labor where abdominal pain may be attributed to uterine activity [6,7]. Recent studies emphasize the importance of integrating clinical findings, hemodynamic trends, and subtle imaging clues when evaluating atypical or localized hemoperitoneum [6,7,8]. A more detailed understanding of this rare entity may aid clinicians in identifying early warning signs and determining the optimal timing of intervention to prevent maternal or fetal compromise [7,8].

Here, we describe a case of massive hemoperitoneum caused by rupture of a superficial uterine fundal vein during preterm labor in a woman with no history of uterine surgery. Fundal venous rupture has been reported only rarely in recent years, making this case a valuable addition to the limited contemporary literature. This report emphasizes that even subtle early symptoms may precede sudden deterioration and that this rare condition should be considered when evaluating unexplained hemoperitoneum in pregnancy.

## 2. Case Report

A 37-year-old gravida 2 para 1 woman at 27 + 0 weeks of gestation presented with a two-day history of left upper quadrant abdominal pain. Her medical history included type 2 diabetes mellitus, and she had no history of uterine or pelvic surgery. On admission, she was hemodynamically stable, with a blood pressure of 112/68 mmHg and a heart rate of 88 beats/min. Mild localized tenderness was noted without guarding or rebound. Hemoglobin was 8.0 g/dL, and fetal heart rate tracing was reactive.

**Figure 1:**
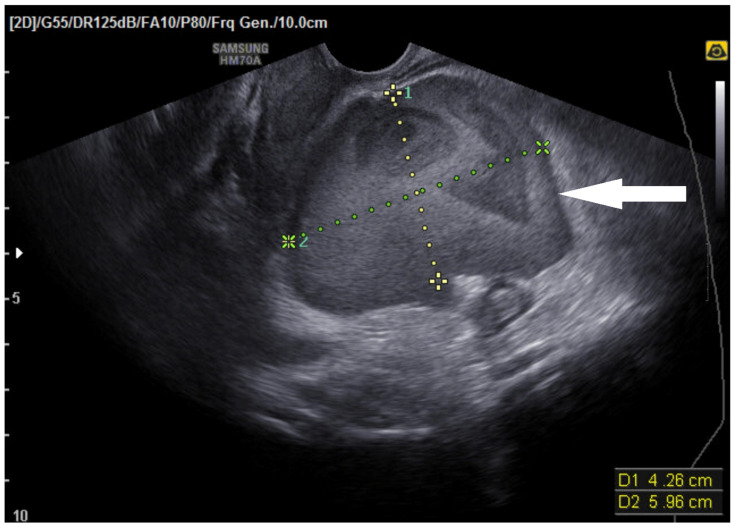
Transabdominal ultrasonography showing a 4.26 × 5.96 cm heterogeneous hematoma adjacent to the left uterine fundus. The arrow indicates the localized hematoma, and the dotted lines represent the measured dimensions.

Transabdominal ultrasonography demonstrated a 4.26 × 5.96 cm heterogeneous lesion adjacent to the left uterine fundus, consistent with a localized hematoma (Figure 1).

**Figure 2:**
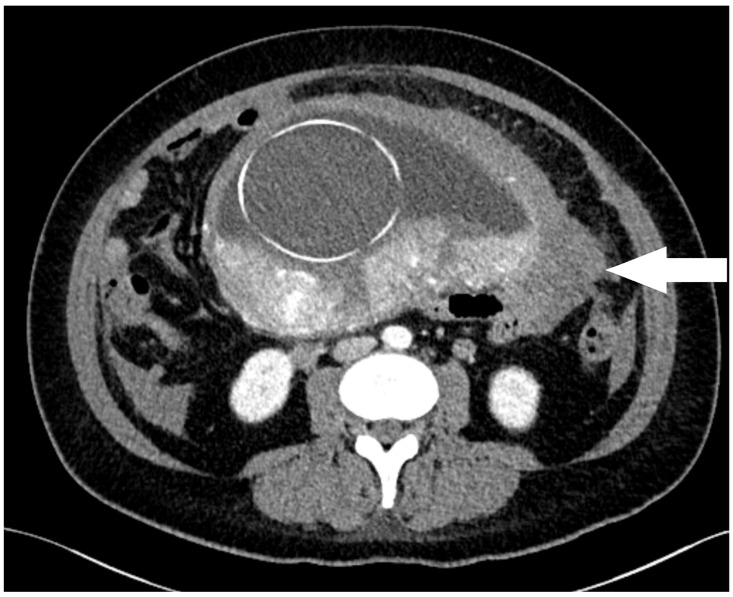
Contrast-enhanced computed tomography demonstrating hemoperitoneum along the left fundal region, with the arrow indicating the localized hematoma without evidence of uterine rupture or active extravasation.

Contrast-enhanced computed tomography further confirmed hemoperitoneum along the left fundal region without evidence of uterine rupture or active contrast extravasation (Figure 2).

Magnetic resonance imaging was not pursued because contrast-enhanced computed tomography was immediately available and provided sufficient information to evaluate hemoperitoneum and exclude active arterial bleeding, and because the patient was experiencing ongoing uterine contractions. Because the patient remained clinically stable and imaging revealed no signs of ongoing hemorrhage, conservative management was initiated. Serial hemoglobin measurements were obtained during conservative management; the initial hemoglobin level was 8.0 g/dL and remained stable at approximately 8.0–8.1 g/dL until a decline to 7.1 g/dL was noted immediately prior to surgery. Uterine contractions occurred every 3–4 min with amplitudes reaching 40–50 mmHg, and intravenous atosiban was administered for threatened preterm labor. Serial hemoglobin measurements were obtained during conservative management, and the hemoglobin level remained stable at approximately 8.0–8.1 g/dL during observation.

**Figure 3:**
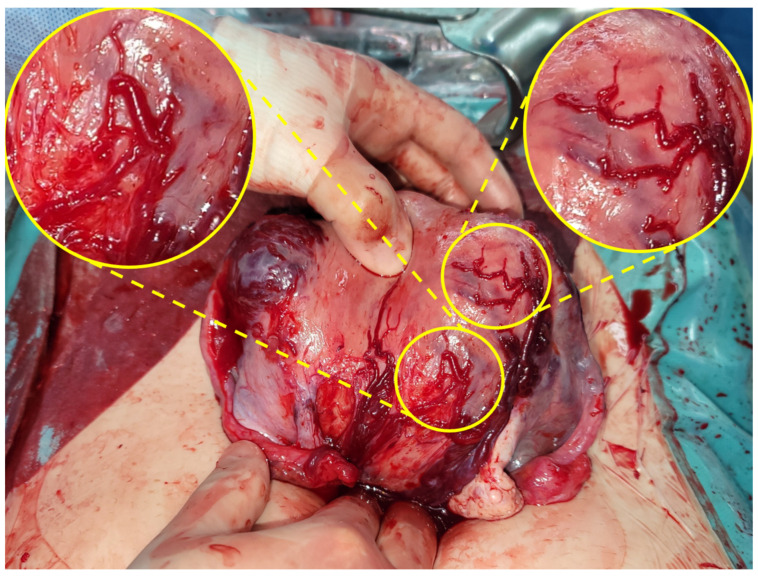
Intraoperative findings showing multiple engorged superficial veins along the left fundal serosa. The inset highlights the superficial venous branch identified as the source of hemoperitoneum.

On hospital day 6 (27 + 6 weeks), she developed sudden worsening of abdominal pain accompanied by dizziness and recurrent fetal heart rate decelerations to 90–120 beats/min. An emergency cesarean delivery was performed for suspected concealed intra-abdominal bleeding or placental abruption. Intraoperatively, approximately 2400 mL of hemoperitoneum was evacuated. The uterus was intact; however, multiple engorged superficial veins were present along the left fundal serosa, and a superficial venous branch demonstrated active bleeding, confirming the source of hemoperitoneum (Figure 3).

No placental abruption or adnexal pathology was identified. Hemostasis was achieved using a fibrin sealant patch. A female neonate weighing 1150 g was delivered with Apgar scores of 3 and 7.

The postoperative course was uncomplicated. Pelvic angiography on postoperative day 2 demonstrated diffuse venous engorgement without active extravasation. Superselective gelfoam embolization was performed prophylactically in the left uterine artery and in abnormally small arterial branches arising from both internal pudendal arteries to reduce residual venous congestion. No post-embolization complications were observed. Follow-up imaging confirmed complete resolution of the hemoperitoneum. The patient was discharged on postoperative day 6 in stable condition. At 3 months postpartum, pelvic computed tomography showed persistent dilation of the left ovarian vein and pelvic venous plexus, suggesting underlying pelvic venous congestion.

This case report was prepared and reported in accordance with the CARE guidelines for case reports.

## 3. Discussion

Spontaneous hemoperitoneum in pregnancy is a rare clinical entity, and its diagnosis remains challenging because early symptoms are often nonspecific and imaging findings may be inconclusive. While uterine rupture, placental abruption, and adnexal vascular accidents are traditionally recognized causes, several recent reports emphasize that rupture of uterine serosal veins represents an underdiagnosed mechanism of intra-abdominal bleeding in pregnancy [1,2,3,4]. Venous structures of the gravid uterus undergo marked physiological changes, including increased venous capacitance, reduced vascular tone, and substantial elevations in uterine perfusion. These hemodynamic alterations may render thin-walled superficial veins particularly susceptible to rupture, especially during episodes of increased intra-abdominal or intrauterine pressure such as preterm labor contractions [3,4,5].

In the present case, the decision to pursue initial conservative management was based on the patient’s hemodynamic stability and the absence of active extravasation on contrast-enhanced CT. Such an approach is consistent with previous reports in which intervention may be deferred when both maternal and fetal parameters remain reassuring [1,2,3,4]. However, the persistence of the fundal hematoma, recurrent uterine contractions, and progressive abdominal discomfort necessitated close clinical reassessment. These evolving findings underscored the dynamic nature of venous-origin hemoperitoneum and ultimately guided the shift from conservative to surgical management once indicators of fetal compromise emerged.

Differential diagnosis of hemoperitoneum in pregnancy is broad and includes uterine rupture, placental abruption, adnexal torsion, vascular anomalies, and rare entities such as hepatic or splenic rupture [1,2,3]. In practice, distinguishing venous-origin hemorrhage from these conditions can be particularly challenging because imaging findings are often subtle, nonspecific, or obscured by gravid anatomy [2,4]. Furthermore, early clinical features—such as abdominal pain or mild hemodynamic changes—frequently overlap among etiologies, increasing the risk of delayed diagnosis [1,2,3,4]. Recognition of characteristic patterns, including localized fundal hematoma and the absence of arterial extravasation on contrast-enhanced imaging, may help guide clinicians toward venous sources of bleeding [3,4,5]. Greater familiarity with these diagnostic pitfalls may assist practitioners in making timely decisions, ultimately improving maternal and fetal outcomes.

Preoperative diagnosis of superficial venous rupture is inherently difficult. Ultrasonography typically demonstrates only a heterogeneous mass or localized fluid accumulation adjacent to the uterus, while computed tomography may reveal hemoperitoneum without active extravasation. As seen in our case, both imaging modalities localized the hematoma to the left fundal region but could not identify the bleeding vessel. These limitations are well documented across case series, in which definitive diagnosis is most frequently made intraoperatively [2,4,9]. Importantly, clinical deterioration can occur abruptly despite initial hemodynamic stability, underscoring the need for heightened suspicion when evaluating patients with unexplained abdominal pain during pregnancy.

The anatomical location in our patient—along the left fundal serosa—is particularly uncommon. Most previously reported venous ruptures involve the posterior uterine wall, often occurring along the posterior uterine surface or adjacent venous plexus [4,5,10]. Previously reported cases of uterine venous rupture during pregnancy are summarized in Table 1 to highlight differences in gestational age, rupture site, management, and outcomes.

As shown in Table 1, most previously reported cases of uterine venous rupture during pregnancy occurred in the third trimester and predominantly involved the posterior uterine wall or broad ligament, frequently in twin pregnancies. In contrast, rupture arising from a superficial vein along the fundal serosa, as observed in the present case, appears to be exceedingly rare. The presence of multiple engorged superficial veins intraoperatively, coupled with persistent dilation of the left ovarian vein and pelvic venous plexus on postpartum imaging, may suggest an underlying tendency toward pelvic venous congestion. This interpretation aligns with emerging evidence describing pelvic venous hypertension, impaired venous drainage, and venous fragility as contributors to vascular rupture in pregnancy [9,11,12,13]. Although pelvic congestion syndrome is typically described in nonpregnant women, hormonal and hemodynamic changes during pregnancy may exacerbate venous distension, increasing the risk of rupture in anatomically dependent regions such as the fundus [11,12,13,14,15].

Previous literature suggests that venous fragility may be compounded by hormonal influences, particularly progesterone-mediated smooth muscle relaxation, which increases venous capacitance and reduces vascular tone [12,14]. Additionally, elevated intrauterine pressure during preterm contractions can create transient peaks in venous backflow, predisposing superficial vessels to sudden rupture [3,4,5]. Although pelvic venous congestion is not routinely evaluated during pregnancy, emerging reports increasingly recognize it as a potential risk factor for spontaneous venous bleeding [9,12]. Although postpartum imaging demonstrated persistent dilation of the left ovarian vein and pelvic venous plexus, these findings should be interpreted with caution, as postpartum imaging cannot definitively confirm the presence or severity of pelvic venous congestion during pregnancy.

Management of spontaneous hemoperitoneum in pregnancy must be individualized based on gestational age, maternal stability, and fetal status. Conservative management may be appropriate in hemodynamically stable patients without imaging evidence of active bleeding; however, most cases ultimately require surgical intervention due to sudden deterioration or fetal compromise [1,4,5]. In our patient, emergent cesarean delivery allowed for rapid evacuation of hemoperitoneum and identification of the bleeding source. The intraoperative pattern of bleeding—slow, diffuse venous oozing—is consistent with previously described cases of uterine serosal vein rupture [2,3].

A unique aspect of this case is the adjunctive use of superselective gelfoam embolization following surgical stabilization, which was performed prophylactically to reduce residual pelvic venous congestion rather than to control active bleeding. Because gelfoam is a temporary embolic agent with spontaneous recanalization, this approach is unlikely to have a significant adverse impact on future fertility, although long-term data remain limited. Although embolization is not routinely used for superficial venous rupture, selective occlusion of abnormal arterial feeders has been reported as a feasible adjunctive therapy in selected obstetric hemorrhage scenarios [16,17]. Given that gelfoam is a temporary embolic agent with recanalization occurring within several weeks, this approach may offer theoretical benefit in reducing persistent venous engorgement while preserving fertility. This decision was further supported by postpartum imaging that demonstrated persistent venous engorgement, suggesting that temporary reduction in pelvic venous pressure could be beneficial. Nevertheless, evidence remains limited, and further studies are needed to clarify the role of adjunctive embolization in spontaneous venous rupture.

This case adds valuable contemporary evidence to the limited literature on superficial uterine venous rupture and highlights the diagnostic and management challenges associated with this condition. It also reinforces the potential relevance of pelvic venous congestion as a predisposing factor. Increased awareness of this entity may facilitate earlier recognition and timely intervention, ultimately improving maternal and fetal outcomes. Therefore, venous-origin hemoperitoneum should be included in the differential when imaging reveals localized hematoma without evidence of arterial bleeding.

This case also raises important considerations for future research. First, it remains unclear which patients are most susceptible to uterine serosal venous rupture, as no standardized risk stratification tools currently exist. Second, the potential association between pelvic venous congestion and spontaneous intra-abdominal bleeding warrants further investigation through prospective imaging studies or postpartum venographic assessments. Finally, the role of adjunctive therapies such as selective embolization, which was used in this case to reduce residual venous engorgement, requires evaluation in larger cohorts to determine efficacy, safety, and fertility outcomes. Continued accumulation of similar cases will be essential to refine diagnostic criteria and optimize future management strategies.

## 4. Conclusions

Spontaneous rupture of a superficial uterine fundal vein is an extremely rare cause of hemoperitoneum in pregnancy and may be difficult to diagnose before surgery. This condition should be considered when pregnant patients present with unexplained abdominal pain or localized hemoperitoneum. Early recognition and prompt intervention are essential for optimizing maternal and fetal outcomes.

## Figures and Tables

**Table 1:** Reported cases of uterine venous rupture during pregnancy.

Author (Year)	Gestational Age	Pregnancy Type	Site of Venous Rupture	Management	Maternal Outcome	Fetal Outcome
Andrés-Orós et al. [10]	37.4 weeks	Twin	Posterior uterine varicose vein	Emergency cesarean delivery with compression hemostasis	Recovered	Both neonates survived
Andrés-Orós et al. [10]	33.6 weeks	Twin	Posterior uterine venous plexus (varicose veins)	Emergency cesarean delivery with venous suturing	Recovered	Intrauterine fetal death of both
Thiam et al. [5]	38 weeks	Twin	Varicose uterine veins on the posterolateral uterine wall	Emergency cesarean delivery with variceal ligation	Recovered	Intrauterine fetal death of both
Maiorana et al. [4]	33 weeks	Singleton	Posterior uterine venous vessels associated with endometriosis	Emergency cesarean delivery followed by hysterectomy	Recovered	Survived
Present case	27 weeks	Singleton	Superficial fundal serosal vein	Cesarean delivery with local hemostasis and adjunctive embolization	Recovered	Survived

## Data Availability

The original contributions presented in this study are included in the article. Further inquiries can be directed to the corresponding author.
